# Does university-industry collaboration improve firm productivity? Evidence from China

**DOI:** 10.1371/journal.pone.0305695

**Published:** 2024-07-26

**Authors:** Yuanqi Li, Zhuo Li, Ting Liu

**Affiliations:** 1 School of Economics, Central University of Finance and Economics, Shahe area, Changping District, Beijing, China; 2 Suzhou Institute of Trade & Commerce, Huqiu District, Suzhou City, Jiangsu Province, China; Inner Mongolia University, CHINA

## Abstract

China’s high-quality development cannot be achieved without high-quality research. As the university is an indispensable source of advanced research, analyzing the impact of university-industry collaboration (UIC) on firm performance helps us understand the significance of universities for China’s economic development and innovation activities. As existing research does not pay attention to the impact of UIC on the productivity of Chinese firms, we examine the impact of such collaboration on firm productivity using natural language processing and by matching China’s intellectual property and listed firms’ operations databases. The empirical results show that UIC can promote firm productivity by improving the quality of their innovations, strengthening internalization efficiency, and broadening their research horizons. Moreover, the UIC process has a pronounced effect on promoting firm productivity in technology- and intellectual property-intensive industries. From the UIC perspective, we interpret China’s economic development and provide new insights for developing countries regarding using universities to alleviate the insufficiency of private R&D investments.

## 1 Introduction

The past two decades have witnessed tremendous economic growth in China, and universities play a significant role in the country’s development. In developing countries such as China, firms with low independent research capabilities have enormous technology demands for external research [1–5]. Therefore, universities are the primary carriers of basic research and, as such, are a significant source for firms to enhance their manufacturing technology [6–11]. Promoting technology transfers from universities to firms could further satisfy and promote firms’ technology demands.

University-industry collaboration (UIC) has been indispensable in transferring knowledge and technology from universities to firms in China [12–18]. The Chinese National Intellectual Property Administration (CNIPA) has spent 1457 million dollars promoting knowledge transfers and stimulating UIC. With incentives from the Chinese government, the number of UIC patents in China will reach more than 760 thousand in 2022, which is expected to increase more than 100 times in the past ten years. Moreover, as per the China Patent Survey Report 2022, published by the CNIPA, the market value of UIC patents is 29.9% higher than that of other patents. Thus, constantly stimulating UIC innovation activities is important for promoting firms’ technology and performance.

Existing research relevant to UIC in China has focused mainly on its impact on firms’ innovation capability and has ignored productivity [14–20]. Thus, based on China’s patent data from 1985 to 2015, we aim to detect whether and how UIC could promote firm productivity. The main result suggested that UIC could significantly promote firm productivity. Based on the findings of the literature, we further interpret the impact of UIC on firm productivity [12–19, 21–23]. First, as innovation quality is an influential factor for firms’ long-term development, the mechanism analysis in this study investigated and suggested that UIC could promote firms’ innovation quality, which is consistent with the existing research [24, 25]. Second, based on firms’ internalization theory, we suggest that UIC could promote the utilization of external knowledge from universities by firms. Finally, the heterogeneity analysis in this study suggested that the impact of UIC on firm productivity becomes more pronounced with increasing distance. As educational resources are unevenly distributed and high-speed rail networks are developed in China [6, 26], geographic distance may be an inconsiderable factor in UIC activities. These results are not only based on the relevant literature to verify the reliability of the conclusion but also provide more prospects for understanding the mechanism through which UIC impacts firm productivity.

We contribute to the literature in three major ways. First, compared with the existing research, we uniquely investigate the impact of UIC on firm productivity. The existing research mainly focuses on the impact of UIC on firms’ innovation capability and ignores its influence on productivity [7, 12–17, 19]. The value of UIC for firm performance is based on something other than the impact of innovation capability, and commercialization in UIC (such as R&D outsourcing) could also directly influence firm productivity. Compared with innovation capability, profit-oriented firms pay more attention to productivity, which directly determines their market performance. Thus, analyzing the impact of UIC on firm productivity could enable a further investigation and evaluation of the significance of universities in China’s economic development.

Second, this study is the first to detect the impact of UIC on firm productivity from the perspective of internalization. Using patent abstracts containing technological details [27–30], this study is the first to use text-mining technologies to measure the degree to which firms internalize university research. This new measurement could identify the efficiency of firms utilizing university research. Using this new measurement, we could resort to internalization and exclude the interference of nontechnological factors (such as universities’ prestige and firms’ strategic innovation behaviors) in the analysis of the impact of UIC on firm productivity. Existing research suggests that firms’ strategic behavior in innovation activities could exaggerate the impact of UIC on firm innovation [27]. Thus, as technology is a remarkable factor in firms’ long-term development, the new measurement allows us to use technology to investigate the impact of UIC on firm development, which is beneficial for detecting the long-term influence of such collaboration.

Finally, given that geographical distance is the core factor in improving UIC activities [18, 27, 31, 32], we further analyze whether geographical distance determines the impact of UIC on firm productivity. The empirical results revealed that UIC at a considerable distance could further strengthen the university’s influence on firm productivity. As education resources are distributed unevenly in China [24, 33], universities at increasing distances from firms could provide more choices for these firms to collaborate, which could further satisfy their technology demands. The empirical results of this study provide a new perspective on geographic distance when comprehending the impact of UIC on firm productivity.

## 2 Literature review

Universities are indispensable for firms in satisfying their technology demands [10–17, 20, 29, 31, 34–38]. By promoting communication between universities and firms, UIC can positively stimulate firms’ R&D activities [37, 38]. Existing research based on firms’ business performance and R&D activities has identified the value of UIC to firm development [29, 36–41]. Ju et al. (2023) used executives’ resumes to measure personnel exchanges between universities and firms and found that these exchanges could promote firms’ innovation capability and efficiency. Muthu et al. (2023) found that knowledge transfer through UIC could promote the quality and quantity of firms’ innovation. Lehmann et al. (2022) also found that UIC could promote firms’ financial performance through knowledge transfers.

According to the relevant research, few studies have analyzed the impact of UIC on a firm’s productivity, and the literature has mainly investigated whether university research could promote a firm’s productivity [11, 23, 28]. The relevant literature based on knowledge spillover and internalization theory reveals the impact of university research on firm productivity. On the one hand, existing studies based on knowledge spillover have shown that UIC can significantly promote firm productivity [1–4, 39, 42]. The relevant literature has shown that knowledge spillover from universities could promote firms’ knowledge management ability [24, 43], strengthen the utilization of external research [2–4, 39–42], and broaden research horizons to improve university research on firm productivity [19, 23]. Lehmann (2022) found that knowledge spillover from universities could promote firms’ financial performance and innovation efficiency, which benefits firm productivity. On the other hand, the existing research has shown that internalization reflects firms’ efficiency in utilizing external research, and that UIC can promote firm productivity through the internalization of external research is not consistent with the existing research [19, 44, 45]. Based on internalization theory, Zahringer et al. (2017) suggested that internalization theory could effectively explain how firms can internalize university research and promote their innovation productivity. However, from a distinct perspective, Myers et al. (2022) noted that firms’ internalization of scientific research is insufficient, and Zhang et al. (2022) and Mowery et al. (2019) noted that deficient innovation capability is a remarkable factor that impedes firms’ internalization capability, which may inhibit their productivity. Thus, the existing research on whether firms can effectively internalize university research is inconsistent.

Our study has three major findings. First, the literature mainly focuses on the impact of UIC on the business performance and innovation ability of firms but ignores its impact on firm productivity [29, 36–41]. Similar to the findings of the relevant research, firms can directly promote their productivity through outsourcing and transferring technology; moreover, given the improvement in intellectual property regulations, this kind of commercialization between universities and firms has become increasingly active [13, 24]. Thus, through firm productivity, the significance of UIC in China’s economic development should be further investigated and evaluated. To compensate for deficiencies in the relevant literature, we use unique patent data to investigate the impact of UIC on firm productivity. Second, in the existing research, whether collaborative universities can promote firm performance through internalization is unclear. The root of this controversy is the lack of appropriate indicators for identifying internalization. The relevant research intuitively, through internalization, analyzes the effect of UIC on productivity; however, more empirical evidence is needed. Thus, based on a text mining technique, we identify the extent to which firms internalize university research and further analyze the impact of UIC on firm productivity. Third, existing studies have shown that geographic distance can promote UIC activities through the knowledge spillover effect [2, 6, 18, 30, 33, 39]. In light of the literature relevant to knowledge spillover, we found that geographic distance is not the main factor that determines the impact of UIC on firm productivity. Our results complement the relevant literature and show that firms can collaborate with cross-regional universities to promote their productivity. According to the relevant literature, the new funding may be relevant because unevenly distributed educational resources and substantial-promoted traffic facilities in China might reduce the traffic and communication costs of geographic distance, and firms could find more universities to collaborate on a broader scope [25, 32]. Thus, this new finding provides a new perspective for understanding how UIC promotes firm productivity.

## 3 Theoretical analysis and research hypotheses

Accordingly, in what follows, we discuss how UIC can promote firm productivity. First, as the quality of a university’s research is higher than that of firms, university research may resort to knowledge spillover effects to promote firms’ innovation quality [24]. In the transfer process, knowledge can be transferred using different carriers (such as articulation and oral presentation), which may enhance innovators’ communications [24, 43]. Thus, firms can utilize knowledge spillover and recombine university research in UIC activities. Chen et al. (2022) suggested that the constitution of research is similar to that of gene segments, and innovators can, through knowledge spillover, recombine external research segments and innovate cutting-edge technology. As research from universities has a higher quality than that from firms, the recombination process of a high-quality knowledge segment is beneficial for creating high-quality research and technology. In addition, as UIC can strengthen the knowledge transfer between universities and firms through frequent communication, it can enhance firms’ innovation quality to fuel productivity [45]. Against this background, we form our first hypothesis:

**H 1**: UIC promotes productivity by enhancing the quality of firm innovation.

Second, internalization theory could explain how UIC promotes a firm’s productivity. Internalization theory regarding UIC refers to the process by which knowledge, expertise, and innovation generated within universities are transferred to and absorbed by firms for commercialization or practical applications [21, 44, 45]. Firms’ internalization efficiency determines the effect of their ability to utilize external research and to promote external research on their technology and productivity [44]. As communication and learning with universities can enhance firms’ internalization efficiency, through UIC activities, firms can absorb external research and enhance their technology and productivity [25]. Specifically, on the one hand, UIC can enhance mutual communication and trust between universities and firms, and promoting relationships between universities and firms can strengthen firms’ internalization of university research [24, 43]. The enhanced trust between universities and firms could facilitate universities’ sharing of core technology and promote university participation [25]. In addition, enhanced mutual communication and trust can help universities cultivate their scientific innovation capability, and UIC can then enhance their internalization of university research. Zahringer (2017) suggested that "face-to-face" communication during UIC activities is conducive to reaching academic consensus among exchangers, increasing the acceptance and recognition of each other’s R&D achievements. Academic consensus could promote communication between universities and firms, which is beneficial for firms to enhance internalization capability. On the other hand, personnel exchange in UIC could promote a firm’s comprehension of scientific research, which is the core factor for firms’ internalization of external research [39, 44]. Ju et al. (2023) and Noack et al. (2021) noted that personnel exchanges can improve employees’ research literacy, thus improving firms’ ability to comprehend scientific research. Against this background, we form our second hypothesis:

**H 2**: UIC promotes firm productivity by strengthening their internalization capability.

Finally, as universities can complement firms in research fields, UIC can promote firm productivity by broadening the research field. Theoretically, universities aiming for exploratory innovation participate in a wide range of research fields, and UIC activities can encourage firms to use wide-field research [7, 24, 39]. On the one hand, Liu et al. (2022) found that profit-oriented firms mainly engage in research related to their products and lack basic research. As cutting-edge technology innovation cannot be separated from basic and diversity-field research, knowledge spillover from diversity-field research in UIC activities can promote firm technology, which benefits firm productivity [7]. On the other hand, firms cannot effectively and individually internalize external knowledge in unfamiliar fields, and frequent communication in UIC could enhance firms’ ability to internalize distinct fields and promote their technology and productivity [24, 39]. Against this background, we form our second hypothesis:

**H 3**: UIC promotes productivity by broadening the breadth of firm research.

## 4 Research design

### 4.1 Modeling

Theoretically, UIC can effectively promote firm production and research activities. This section further evaluates the impact of UIC on firm productivity through the use of patent data via empirical analysis. This chapter constructs the benchmark model shown in Eq (1).

$$TFP_{it+2}=\alpha_{0}+\alpha_{1}*WhtCoPat_{it}+\beta *Controls_{it}+\mu_{i}+\xi_{t}+\eta_{it},$$ (1)

where *i* represents the index of firms and *t* represents the index of years, and the dependent variable (*TFP*_*it*+2_) is the total factor productivity (TFP) of listed company *i* in year *t* + 2. Subsequently, the benchmark model analyzes the impact of UIC on firm productivity over a two-year horizon. The independent variable of UIC is *WhtCoPat*_*it*_, which indicates whether firm *j* collaborated with a university in year *i*. *α*_0_ is a constant term, and *α*_1_ is the coefficient of the core variable to be estimated in this study, which reflects the impact of UIC on firm productivity. The vector *Control*_*it*_ is a set of firms *i*’s characteristic controls defined in the following section. In various specifications, we include year (*ρ*_*t*_) and firm (ϵ_*i*_).

### 4.2 Variable construction

#### Dependent variables

The dependent variable is Chinese listed firm productivity. Following Lu and Lian (2012), we base this study on the asset-liability and business operation databases from China Stock Market & Accounting Research (CSMAR) and uses the LP and OP methods to measure the TFP of listed firms in different years.

#### Independent variables

The independent variable in this study is UIC. On the one hand, this independent variable is whether the university and firm collaborated in different years (WheCoPat). On the other hand, we also measure the scale of UIC and detect its impact on a firm’s productivity to ensure the robustness of the conclusions of the empirical analysis. The scale of collaboration uses the number of collaboration patents (NumCoPat). To classify patent applicants, we employ measures based on Hong et al. (2013) to divide patent applicants into universities, firms, individuals, and hospitals. Accordingly, we define patents whose applicants include universities and firms as UIC patents. Thus, we identify whether listed companies have cooperated with universities in different years and measure the scale of UIC using the number of collaborative patents each year.

#### Mechanism variables

The mechanism variables in this study are the firm’s innovation scale and quality, its internalization efficiency, and its research breadth. Regarding the scale and quality of corporate innovation, we follow Fang et al. (2020) in measuring the scale of corporate innovation using the number of invention patents filed by listed companies (InvPatNum) and the total number of patents (PatNum). In contrast, we draw on Byun et al. (2021) and Arora et al. (2021) to measure the quality of firms’ patents by using the number of citations cited by other invention patents per year (NumInvCita) and the total number of citations cited by a variety of patents (NumCita). Regarding the internalization efficiency of firms, we follow Hain et al. (2022), who measured the degree to which firms absorb knowledge from universities through text analysis methods by using the abstract text of invention patents. First, we use the Ali OpenAI platform to construct the word segments of patent abstracts. Subsequently, we eliminate stop words based on the stop-word dictionary released by the Harbin Institute of Technology. Second, we use the TF-BIDF method to construct word vectors based on the word segments of the patent abstract, following Kelly et al. (2021), to avoid the influence of the patent application year. Finally, based on the word vector of the patent abstracts, the extent to which firms internalize the research of universities is recognized by the backward similarity measurement [46, 47]. Specifically, this measure is calculated as follows:

$$BS_{i}^{\tau }=\sum_{j\in \xi_{i\tau }}\rho_{ij},$$ (2)

$$lnSPProv_{it}=\log\left(BS_{i}^{\tau }+1\right),$$ (3)

where *i* represents the index of listed firms and *j* represents the index of universities. *ρ*_*ij*_ denotes the similarity between the abstract of firm *i*’s patents and university *j*’s patents, and ξ_*iτ*_ is the set of universities in the same province as firm *i*. Moreover, the calculation of patent back similarity only considers firms’ patents released within τ years of those released by universities. Considering that a certain lag exists between the publication of university patents and their absorption by firms, the window period indicates the process of firms absorbing university research. Following Kelly et al. (2021), we use the window period (τ) for constructing backward similarity as five years. Eventually, we define the efficiency of firms absorbing university results as $BS_{i}^{\tau }$, and Eq (3) shows its logarithmic form. Regarding firms’ research breadth, we follow Byun et al. (2021) and use the patent IPC classification number (NumIPC) to measure firms’ research breadth.

#### Heterogeneous variables

The heterogeneity variables classify the number of collaborative patents based on the distance between universities and firms. We use the longitude, latitude, province, and city of a university and firm to identify the UIC density under different scopes. On the one hand, according to Yi et al. (2021), we use longitude and latitude to calculate the great circle distance between universities and firms. The latitude and longitude information of listed firms is obtained from the CSMAR database, and university information is analyzed using the Baidu map API platform. Furthermore, we used the Tencent map API platform to revise universities’ latitude and longitude information, and the samples with inconsistent information from those two platforms are queried manually. Accordingly, we divide the distances between the collaborating universities and firms into (0, 100), (100, 200), (200, 300), (300, 400), (400, 5000), and (500, +), all in kilometers. Then, we measure the number of patents in different UIC ranges. On the other hand, we categorize UIC into same-province, same-city, different-province, different-city, and same-province-different-city UIC based on information about the province and city where the collaborating university and firm are located.

#### Control variables

Referring to Byun et al. (2021) and Arora et al. (2021), we control for listed company-level information in the empirical analysis, including firm cash flows (*Cash*_*it*_), firms’ R&D investment stock (*R*&*D Stock*_*it*_), firms’ net fixed assets (*Asset*_*it*_), and total corporate assets (*Caputal Stock*_*it*_). Moreover, the covariance test was conducted by applying the variance inflation factor to all of the variables. The results reveal that no severe covariance problem exists.

### 4.3 Data sources and descriptive statistics

In this study, Chinese listed companies from 2007 to 2015 were selected as the research sample. The information and data were collected from the quarterly financial reports of listed Firms in the CSMAR Database. Moreover, the patent data of firms and higher education institutions were obtained from the China Intellectual Property Office, which were available from 1985 to 2015. In addition, basic information on higher education institutions was collected from the Ministry of Education of China. We also complement universities’ latitude, longitude, provinces, and cities through the Baidu and Tencent map API platforms. According to the need, samples with missing variables were eliminated from the database matching process. Finally, 15239 observations from 2800 firms, which are unbalanced panel data, were obtained. Table 1 presents the descriptive statistics of the dependent variables, independent variables, mechanism variables, heterogeneity variables, and control variables, which include information on the variables’ sample sizes, means, and variances.

**Table 1:** Descriptive statistics.

Variable symbol	Variable meaning	Number of observations	Mean	SD	Minimum	Maximum
*OP-TFP_it_*	TFP of listed firms (OP method)	15239	3.706	0.85	−1.6417	8.0704
*LP-TFP_it_*	TFP of listed firms (LP method)	21118	7.9035	1.0971	4.7184	11.8594
*WHP_it_*	Whether the listed company cooperates with universities	24841	1.326	0.4688	1	2
*OwnTolPat_it_*	Number of patents filed by listed companies	24842	13.6628	123.6368	0	6381
*OwnCrPat_it_*	Number of invention patents filed by listed companies	24842	6.3457	90.3685	0	5821
*CitaTol_it_*	Number of citations received from patents of listed companies	24362	60.6475	486.7463	0	25447
*CitaInv_it_*	Number of citations received from invention patents of listed companies	24677	44.5659	439.9541	0	24657
*AcmCityTol_it_*	Ability of listed companies to absorb and utilize the same cities universities’ research	24841	849.8205	19876.22	0	1584583
*AcmProvTol_it_*	Ability of listed companies to absorb and utilize same-province universities’ research	24841	947.8269	20339.35	0	1598676
*NumTolIPC_it_*	Number of distinct IPCs of firms’ patents	24843	3.8013	11.2777	0	241
*NumCrIPC_it_*	Number of distinct IPCs of firms’ invention patents	24843	2.0614	2.0614	2.0614	2.0614
* $NumPat_{it}^{0-100}$ *	Number of UIC patents each year applied by listed companies within 100 km	24843	0.0066	0.1731	0	12
* $NumPat_{it}^{100-30}$ *	Number of UIC patents applied by listed companies each year in the 100–300 km range	24843	0.0095	0.172	0	10
* $NumPat_{it}^{300-50}$ *	Number of UIC patents each year applied by listed companies in the 300–500 km range	24843	0.0048	0.129	0	10
* $NumPat_{it}^{500+}$ *	Number of UIC patents each year applied by listed companies with universities 500 km away	24843	0.09	0.8627	0	30
*CityPatNum_it_*	Number of UIC patents applied by listed companies in cooperation with the same-city universities	24843	0.0043	0.1535	0	12
*NonCityPatNum_it_*	Number of UIC patents applied by listed companies in cooperation with universities with distinct-city universities	24843	0.1066	0.9369	0	36
*ProvPatNum_it_*	Number of UIC patents applied by listed companies in cooperation with same-province universities	24843	0.0074	0.1881	0	12
*NProvPatNum_it_*	Number of UIC patents applied by listed companies in cooperation with distinct province universities	24843	0.1036	0.9142	0	30
*ProvNcityPatNum_it_*	Number of UIC patents applied by listed companies in cooperation with same-province-distinct-city universities	24843	0.0031	0.1087	0	10

### 4.5 Identification strategy

We explore the impact of UIC on firms’ TFP. In general, omitted variables and reverse causality problems affect the empirical analysis process. Thus, we construct a stringent control specification and employ the instrumental variable (IV) method to circumvent the endogeneity problems generated by the model. First, according to existing studies, firms’ absorption and reorganization of external knowledge take time [11, 28]. Thus, we focus this study’s empirical analysis on the impact of UIC on firm productivity with a two-year lag. Hence, the likelihood of a reverse causality problem in the model is extremely low, and UIC decisions are unlikely to depend on a firm’s future production levels. Second, we control for the additional fixed effects of the industry, city, province, firm-industry, firm-province, and firm-city interaction terms, in addition to the original control variables and fixed effects to further mitigate the omitted variable problem. The extra control variables could exclude the influence of omitted variables at the industry and regional levels to induce mutual effects on university and firm decisions. Finally, we further mitigate the endogeneity problem via the IV method. Following Bloom et al. (2013), we construct IVs using the policy of the "Provincial-Ministry Jointly Built State Key Laboratory Cultivation Bases" (PM-JBSK Laboratory Bases) promulgated by the Ministry of Science and Technology (MST). Thus, we used the number of patents filed by universities participating in the policy and located around listed companies to construct the IVs as follows:

$$IV_{it}=\sum_{u\in D_{i}}NumPat_{u,t}*Policy_{u,t}.$$ (4)

In Eq (4), *D*_*i*_ is the set of universities participating in the PM-JBSK Laboratory Bases policy within 50 km of firm *i*, and *NumPat*_*u*,*t*_ is the number of patents of universities *u* applied to in year *t*. Moreover, *Policy*_*u*,*t*_ is a dummy variable that captures whether university *u* participates in the PM-JBSK Laboratory Bases policy in year *t*. IV reflects the density of universities engaged in scientific research on listed companies and meets the requirements of exogeneity and relevance. First, we use the number of patents applied for by universities around listed companies to construct the IVs. The literature has demonstrated that the density of universities around firms increases the probability of UIC [21]; therefore, IVs satisfy the requirement of relevance. On the other hand, the MST and provincial science and technology departments jointly sponsor the PM-JBSK Laboratory Bases policy to enhance basic scientific research from local universities and research institutes. Generally, the policies described by the provincial ministries are not influenced by individual firms; thus, this IV is externalized to the business activities of local firms.

## 5 Empirical results and analysis

### 5.1 Baseline regression

Table 2 presents the OLS regression results of the baseline model, shown in Eq (1), which uses the fixed-effects model with standard errors clustered at the firm level. In columns (1)–(4) of Table 2, the coefficients of the independent variables of each model tend to stabilize as control variables are gradually added. All of the coefficients are significantly positive at the 5% level. The empirical results strongly suggest that UIC effectively promotes firm productivity.

**Table 2:** Effect of UIC on firm productivity.

	(1)	(2)	(3)	(4)
Variant	*TFP_it+2_*	*TFP_it+2_*	*TFP_it+2_*	*TFP_it+2_*
*WthCoPat_it_*	0.1082**	0.1435**	0.1435**	0.1434**
	(0.0365)	(0.0367)	(0.0367)	(0.0367)
*N*	15109	14734	14734	14734
*Adj R^2^*	0.4692	0.4828	0.4827	0.4827
Controls	NO	NO	NO	YES
Year fixed effects	YES	YES	YES	YES
Firm fixed effect	YES	YES	YES	YES

Note: ***, **, and * indicate significance at the 1%, 5%, and 10% levels, respectively; robust standard errors clustered at the firm level are in parentheses. The following tables are identical. The results for each control variable are not provided due to the article’s length but are available to interested readers on request. Only year and firm fixed effects are controlled for in column (1). Column (2) reflects the addition of firm R&D investment stock (in millions) and firm fixed assets (in millions) as control variables in addition to controlling for year and firm fixed effects. Column (3) reflects the addition of firm R&D investment stock (in millions), firm fixed assets (in millions), and firm operating cash flow (in millions) as control variables. Finally, column (4) reflects the addition of firm R&D input stock (millions) and firm fixed assets (millions) as control variables and firm operating cash flow (millions), and firm cash flow (millions) as fixed effects on top of the control year and firm fixed effects.

### 5.2 Robustness tests

#### Adjusting the explanatory variable measures

We measure the productivity of firms through the LP method to test the robustness of the conclusions of the benchmark regression. Table 3 presents the empirical results. Among them, the core variables in column (1) are significantly positive at the 5% level, suggesting that the conclusions on the effect of UIC on the promotion of firm productivity are relatively robust after excluding the influence of methods used to measure firm productivity.

**Table 3:** Robustness analysis.

	(1)	(2)	(3)	(4)	(5)	(6)	(7)	(8)
Variable	*TFP_it+2_*	*TFP_it+1_*	*TFP_it+2_*	*TFP_it+2_*	*TFP_it+2_*	*TFP_it+2_*	*TFP_it+2_*	*TFP_it+2_*
	LP method	OP method	OP method	OP method	OP method	OP method	OP method	OP method
*WtCoPat_it_*	0.1185**	0.0721**	0.1365***	0.1250***	0 .1483***	0.1481***	0.1416***	0.1450***
	(0.0564)	(0.0392)	(0.0357)	(0.0379)	(0.0374)	(0.0370)	(0.0368)	(0.0368)
*N*	13,921	14,734	14,723	14,222	14,705	14,683	14,734	14,712
*Adj R^2^*	0.5330	0.4879	0.4008	0.3741	0.4741	0.3630	0.4829	0.3633
Year fixed effects	Yes	Yes	Yes	Yes	Yes	Yes	Yes	Yes
Firm fixed effect	Yes	Yes	Yes	No	Yes	No	Yes	No
Industry fixed effect	No	No	Yes	No	No	No	No	No
Industry * firm fixed effects	No	No	No	Yes	No	No	No	No
Urban fixed effect	No	No	No	No	Yes	No	No	No
Urban * firm fixed effects	No	No	No	No	No	Yes	No	No
Province fixed effects	No	No	No	No	No	No	Yes	No
Province * firm fixed effects	No	No	No	No	No	No	No	Yes

#### Adjustment of firms’ business performance in different years

Existing studies have concluded that UIC has a lagged effect on promoting firm production processes; however, the lag period is set differently [9, 11, 12, 28]. Therefore, to test the robustness of the benchmark model, in this section, we replace the lag period. According to columns (2) Table 3, UIC significantly contributes to firm productivity with one lag. Therefore, the lag period does not affect the promotion effect of UIC on firm productivity, and the conclusions of the benchmark model are relatively robust.

#### Controlling for fixed effects at the industry, provincial, and municipal levels

UIC may be affected by regional- and industry-level factors. To test the robustness of the benchmark model, we further control for regional- and industry-level fixed effects. First, columns (3) and (4) of Table 3 control for industry and industry-firm fixed effects, respectively, and the coefficients on the independent variables remain significantly positive at the 1% level. Second, columns (5) and (6) of Table 3 control for city and city-firm fixed effects, respectively. Moreover, the parameters of the independent variable are significantly positive, suggesting that UIC can significantly promote firm productivity. Finally, columns (7) and (8) of Table 3 control for the firm’s province and province-firm fixed effects, respectively. Furthermore, the empirical results show that the independent variables are positive at the 1% significance level. Therefore, the findings of this study are consistent with the benchmark model after excluding the influence from the industry, city, and province levels, and the model has remarkable robustness.

### 5.3 Endogenous issues

Table 4 demonstrates the results of the two-stage regression of the IV method. In this study, the number of patents filed by participating policy universities within 50 and 150 km were selected to construct the IVs, ensuring the robustness of the empirical results. In the first-stage regression with two different IVs, the Cragg-Donald Wald F value and Kleibergen‒Paap Wald F value are more significant than the empirical value of 10. This finding indicates that underidentification and weak IV problems do not plague the empirical conclusions. Columns (1)–(3) and (4)–(6) use the number of patents applied for by universities within 50 km and 150 km of the firm, respectively, to construct the IVs. According to the results of the second-stage regression of IVs in columns (2) and (5), the coefficient of UIC is significantly positive at the 5% level. Thus, the empirical results of the two-stage regression are consistent with the baseline regression results, indicating that UIC can significantly promote firm productivity.

**Table 4:** Instrumental variable analyses: Effect of UIC on firm productivity.

Impact distance	50	50	50	150	150	150
	1SLS	2SLS	Reduce Form	1SLS	2SLS	Reduce Form
Variant	*WHP_it_*	*TFP_it+2_*	*TFP_it+2_*	*WHP_it_*	*TFP_it+2_*	*TFP_it+2_*
*LnPlNumPatUnv50_it_*	0.0000047**		0.000027***	0.000005***		0.0000213***
	(0.0000019)		(0.0000072)	(0.0000015)		(0.0000054)
* $\hat{WHP_{tt}}$ *		5.8167**			4.3160**	
		(2.6761)			(1.6534)	
*N*	14,734	14,734	14,734	14,734	14,734	14,734
*Adj R^2^*	0.6067	-1.5214	0.4830	0.7029	-2.8149	0.4832
*Control variable*	Yes	Yes	Yes	Yes	Yes	Yes
*Year fixed effects*	Yes	Yes	Yes	Yes	Yes	Yes
*Individual fixed effect*	Yes	Yes	Yes	Yes	Yes	Yes

## 6 Further analysis

### 6.1 Mechanism analysis

#### Impact of UIC on the scale and quality of firm innovation

Compared with universities, firms play a leading role in patent creation; however, their innovation quality has an observable gap with that of universities. Given the difference between university and firm innovation, we further examine the impact of UIC on firms’ innovation quality and quantity. Moreover, we identify the quantity and quality of firms’ innovation via the number of firm-applied patents and citations that firm-applied patents receive each year. According to columns (1) of Table 5, the empirical results reveal that the independent variables are nonsignificant, and we conclude that UIC does not affect the quantity of firm innovation. In addition, according to columns (2) of Table 5, UIC is significantly positive at the 5% level. Thus, we further conclude that UIC contributes considerably to the quality of firm innovation. This empirical result proves Hypothesis 1, and UIC could effectively enhance firm productivity by increasing innovation quality.

**Table 5:** Mechanism analyses: Effects of UIC on the scale and quality of innovation in universities.

	(1)	(2)
Variant	*lnPatNum_it+2_*	*lnNumCita_it+2_*
*WthCoPat_it_*	0.0351	0.0771**
	(0.0470)	(0.0341)
*N*	7,687	15,155
*Adj R^2^*	0.7848	0.7329
Year fixed effects	Yes	Yes
Firm fixed effect	Yes	Yes

#### Impact of UIC on firm efficiency in absorbing knowledge achievements from universities

We analyze the impact of UIC on firms’ internalization capability (Table 6). According to the empirical results in column (1), UIC is significantly positive at the 1% level, indicating that UIC can encourage firms to absorb universities’ scientific research in the same city range. In addition, the coefficient of the independent variable in column (2) is significantly positive at the 1% level, indicating that UIC can encourage firms to absorb the results of universities in the same province. Thus, the empirical results in Table 6 support Hypothesis 2, and UIC can enhance firm productivity by improving their internalization ability.

**Table 6:** Mechanism analyses: Effects of UIC on firms’ absorption capability.

	(1)	(2)
Variant	*lnAcmSPCity_it+2_*	*lnAcmSPProv_it+2_*
*WthCoPat_it_*	0.2540***	0.2644***
	(0.0970)	(0.1012)
*N*	14,167	14,167
*Adj R^2^*	0.7310	0.7260
Year fixed effects	Yes	Yes
Firm fixed effect	Yes	Yes

#### Impact of UIC on firms’ breadth of research fields

Existing studies have argued that UIC expands their research fields and promote firms’ technological reorganization, which can promote innovation activities and enhance productivity [8, 27, 48]. Hence, we identify the research scope of firms using their issued patents’ IPC classification codes (three-digit codes). Because firms are mainly committed to the creation of applied research, we measure their research scope using the number of distinct IPCs from design and utility patents. Table 7 shows the empirical results. As shown in columns (1) and (2), the UIC variable is significantly positive at the 1% level. Thus, UIC can broaden the field, and this empirical result supports Hypothesis 3. Therefore, in the UIC process, universities broaden firms’ research fields, promoting their productivity.

**Table 7:** Mechanism analyses: Effects of UIC on the width of firms’ research fields.

	(1)	(2)
Variant	*ln(NumDsPatIPC_it+2_)*	*ln(NumUtPatIPC_it+2_)*
*WthCoPat_it_*	0.3335***	2.3372***
	(0.1641)	(0.4446)
*N*	14,167	14,167
*Adj R^2^*	0.6320	0.7367
Year fixed effects	Yes	Yes
Firm fixed effect	Yes	Yes

### 6.2 Heterogeneity analysis

Existing studies have shown that the intensities of intellectual property and technology in different industries are significantly distinct, indicating that industries’ technology demands vary significantly. We explore the effect of UIC on firm productivity in different industries. We classify the industries into intellectual property- and technology-intensive industries according to the industry divisions published by the National Bureau of Statistics. Research activities are more active in intellectual property- and technology-intensive industries than in other industries. The scale of R&D capital and personnel investments is also larger [47]. Specifically, firms in technology- and intellectual property-intensive industries employ more than three times more R&D personnel than do those in other industries. Similarly, R&D investment is more than twice as large as that in other industries. Considering the heterogeneity of R&D activities and technological dependence in various industries, we analyze the impact of UIC on firm productivity in different industries through empirical methods. The empirical results in columns (1) and (2) of Table 8 reveal that UIC can significantly promote the productivity of firms in technology- and intellectual property-intensive industries. The independent parameters are significantly positive at the 1% level. Furthermore, according to columns (3) and (4) of Table 8, the coefficients of UIC are not significant; thus, UIC does not significantly affect the productivity of firms in non-technology- and nonintellectual property-intensive industries. In this regard, the different technological demands of firms in different industries may lead to differences in the effect of UIC on the promotion of firm productivity. That is, the effect of UIC on the promotion of firm productivity in technology- and intellectual property-intensive industries with high technological needs is greatly pronounced.

**Table 8:** Heterogeneity analyses: Effect of UIC on firm productivity in different industries.

	(1)	(2)	(3)	(4)
	Nontechnology intensive	Technology-intensive	Nonintellectual property-intensive	Intellectual property intensive
Variant	*TFP_it+2_*	*TFP_it+2_*	*TFP_it+2_*	*TFP_it+2_*
*WhtCoPat_it_*	0.0649	0.1675***	0.0215	0.1969***
	(0.0830)	(0.0552)	(0.0607)	(0.0453)
*N*	2,454	4,018	2,454	8,091
*Adj R^2^*	0.3694	0.3044	0.6487	0.3266
Year fixed effects	Yes	Yes	Yes	Yes
Firm fixed effect	Yes	Yes	Yes	Yes

Because of the uneven distribution of educational resources and the rapid development of infrastructure in China, firms are no longer limited to cooperating with local universities. Instead, they cooperate with institutions with suitable technology and creation. Hence, we analyze the impact of cross-regional UIC on firm productivity. Table 9 presents the empirical results. On the one hand, column (1) of Table 9 shows that the coefficient of the number of collaboration patents within 500 km of the collaborating firm is insignificant. However, the number of collaboration patents outside the range of 500 km is significant at the 5% level. Thus, we conclude that long-distance UIC plays a significant role in promoting firm productivity. However, close-distance UIC has no significant impact on firms’ technological levels or productivity. Moreover, with its narrow research scope, this empirical result suggests that local universities cannot satisfy firms’ technology demands and that investment in local UIC may not significantly improve firms’ innovation and manufacturing technology. On the other hand, we further analyze the impact of administrative distance on UIC efficiency. According to the results in columns (2) and (3) of Table 9, we determine that the scale of UIC patents within the same city of a firm does not significantly impact its productivity. However, the scale of cross-city UIC patents can significantly increase a firm’s productivity. In addition, columns (2) and (4) show that the impact of UIC on the productivity of same-province firms is insignificant. However, the coefficient of cross-provincial cooperation is significantly positive at the 5% level. Thus, we suggest that this case may be related to the uneven distribution of educational resources in China and that finding suitable universities to collaborate in innovation in the local region is difficult for firms. Hence, local cooperation has a limited effect on promoting firms’ production technology. Hong et al. (2013) also demonstrated that local governments promote local UIC. This kind of involuntary collaboration may increase firms’ ineffective investments and inhibit the effect of UICs on promoting firms’ production and R&D activities. Innovation from involuntary collaboration can satisfy firms’ technology demands. Therefore, in causal analysis, with the decreasing influence of distance, making sure that the effects of UIC on firm productivity should be to pay more attention to local universities’ technology supply and firms’ demands than to the communication cost between universities and firms.

**Table 9:** Heterogeneity analyses: Effect of distinct distance UIC on firm productivity.

	(1)	(2)	(3)	(4)
Variant	*TFP_it+2_*	*TFP_it+2_*	*TFP_it+2_*	*TFP_it+2_*
* $\text{ln}\left(NumCoPat_{it+1}^{0-100}\right)$ *	0.1743			
	(0.1290)			
* $\text{ln}\left(NumCoPat_{it+1}^{100-300}\right)$ *	0.0942			
	(0.0891)			
* $\text{ln}\left(NumCoPat_{it+1}^{300-500}\right)$ *	−0.1473			
	(0.1707)			
* $\text{ln}\left(NumCoPat_{it+1}^{500+}\right)$ *	0.0874**			
	(0.0406)			
* $\text{ln}\left(NumCoPat_{it+1}^{City}\right)$ *		0.0356	0.0333	
		(0.1441)	(0.1458)	
* $\text{ln}\left(NumCoPat_{it+1}^{ProvNonCity}\right)$ *		0.0657		
		(0.1863)		
* $\text{ln}\left(NumCoPat_{it+1}^{NonProv}\right)$ *		0.0877**		0.0877**
		(0.0369)		(0.0370)
* $\text{ln}\left(NumCoPat_{it+1}^{NonCity}\right)$ *			0.0853**	
			(0.0362)	
* $\text{ln}\left(NumCoPat_{it+1}^{Prov}\right)$ *				0.0541
				(0.1247)
N	14,734	14,734	14,734	14,734
Adj R^2^	0.4822	0.4821	0.4821	0.4821
Year fixed effects	Yes	Yes	Yes	Yes
Firm fixed effect	Yes	Yes	Yes	Yes

## 7 Discussion

Recently, researchers studying China’s innovation noted that firms’ R&D activities could not sufficiently satisfy the technology demand from manufacturing activities [26, 49–54]. However, the main results of this study show that UIC can significantly promote firm productivity. The results of the mechanism analysis and those in Table 6 show that UIC can assist firms with internalizing external research. Thus, UIC activities can, through absorbing and transforming external research, satisfy firms’ technology demand from product manufacturing activities. Existing research shows that UIC has distinct ways of influencing firms’ performance, such as through university reputations and innovation outsourcing [19, 55–57]. Among them, technology internalization is one way to promote firms’ long-term development, among many forms of influence. Muthu et al. (2023) and Zahringer et al. (2017) suggested that firms’ internalization capability could further promote their ability to comprehend external research and apply research to update their manufacturing technology. Thus, firms’ internalization capability is the core factor that explains the positive effect of UIC on firm productivity. Therefore, although promoting knowledge transfer from universities to firms is important for firms to satisfy their technology demands, strengthening firms’ internalization capability in UIC activities can determine whether firms can utilize and transform external research. Internalization capability is the crucial factor for firms’ long-term development.

In addition, in light of existing research, this study’s heterogeneity analysis reveals that geographical proximity is not an indispensable factor in determining the positive effect of UIC on firm productivity. Specifically, the empirical results reveal that UIC at a long distance could significantly promote firm productivity. Considering the results of this study with those of existing research shows that China’s educational resources are distributed unevenly, and firms cannot find proper, local universities to collaborate with [33, 58–63]. In addition, Hong et al. (2013) found that local governments prefer to promote local UIC activities and that undesirable collaboration may increase firms’ undesirable investments. Thus, compared with communication costs, technical adaptation in collaboration might be more remarkable in promoting the value of UIC in China’s economic development.

The findings have several policy implications. On the one hand, regulations should consider research externalities. As research externalities encroach on profits from firms’ R&D activities, even with substantial subsidies, firms have insufficient motivation to devote themselves to research. Thus, as university researchers have public characteristics, policies could subsidize universities’ research and resort to knowledge transfer from universities to firms to address firms’ research deficiencies. In this way, policy should strengthen firms’ internalization efficiency, such as by encouraging the planning of entrusted training of employers. Internalization efficiency determines whether knowledge can be transferred to firms and applied to their manufacturing activities. On the other hand, geographic proximity is not a core factor that determines the positive effect of UIC on firm productivity but is a remarkable factor in encouraging UIC activities. As firms can find more suitable universities with which to cooperate within a broader range, improving the traffic infrastructure, especially for long-distance transportation, could strengthen the positive effect of UIC activities on firm productivity.

## 8 Conclusion and limitations

In the introduction, we noted that the relevant literature mainly analyzes the impact of UIC on firms’ innovation capability to interpret the value of university research on China’s economic development. Compared with innovation activities, profit-driven firms pay more attention to business performance. Thus, we further investigate the impact of UIC on firm productivity. According to patent data from 1985 to 2015, we use a fixed effect model and conclude that UIC could promote firm productivity. Our results strengthen the value of university research from the perspective of firm productivity. The main conclusion in our study could complement existing research deficiencies through the firm productivity perspective and further evaluate UIC’s significance for China’s economic development.

The mechanism analysis reveals that UIC can, through internalization efficiency, promote firm productivity. Internalization efficiency significantly determines the impact of UIC activities on firms’ production technology. As technology has been a core factor influencing China’s economic restructuring, enhancing firms’ ability to utilize their technology and research has been a core agenda of the UIC policy system. Moreover, our empirical results reveal that UIC can promote firms’ innovation quality and broaden their research field. Those results prove that UIC can benefit long-term firm development, which could encourage China in reforming high-quality productive forces. Moreover, we reveal that UIC can more effectively promote firm productivity in intellectual property- and technology-intensive industries. Therefore, the investigation of UIC policy should consider the differences in technology demands among firms’ industries. Finally, compared with local UICs, long-distance UICs are more effective at promoting firm productivity. On the one hand, the uneven distribution of education resources in China means that firms cannot find the appropriate local university to collaborate with. On the other hand, China’s developed rail system reduces the cost of long-distance communication. Thus, as the traffic facility reduces regional communication costs, China could resort to the developed railway system to promote firms’ regional UIC, which could enable firms’ external resources to flourish and simulate firms’ R&D activities.

This study has several limitations and suggests topics for several relevant future works, which must be explained carefully. (1) Although using a patent’s author to identify UICs is the most frequently used method, patent transfer and licensing can also reflect UIC activities. Thus, future work should collect different kinds of datasets and consider different methods to identify UIC activities and detect the impact of UIC on firm productivity. An extra indicator of UIC can not only ensure the robustness of the empirical analysis but also provide additional perspectives for analyzing the mechanism of the impact of UIC on firm performance. (2) We use IV methods and stricter controlling variables to address the endogeneity problem. However, in future works, more methods, such as spatial econometric models, are needed to further exclude the interference of missing variables and further reverse the causality problem. (3) The empirical results of the heterogeneity analysis revealed that geographical distance is not an influential factor in determining the impact of UIC on firm productivity. Although technical adaptability may influence the impact of UIC on firm productivity, further empirical research is needed to verify this perspective. In addition, a need also exists for sophisticated mechanism analysis to explain the impact of geographical distance on China’s UIC activities.

## Supporting information

S1 File Data (ZIP)
